# Characterization of near-complete human *Pegivirus* 2 (HPgV-2) genomes in individuals co-infected with hepatitis C virus (HCV) in Cameroon

**DOI:** 10.1128/mra.00069-26

**Published:** 2026-03-12

**Authors:** Aristide Mounchili-Njifon, Vireak Heang, Leakhena Pum, Loique Landry E. Messanga, Moise Henri Moumbeket-Yifomnjou, Abdou Fatawou Modiyinji, Desmon Toutou Toutou Tsafack, Ladifatou Nzi Mbouo-Njoya, Simon Frederic Lissock, Pretty Rosereine Mbouyap, Jean Paul Assam Assam, Erik A. Karlsson, Janin Nouhin, Richard Njouom

**Affiliations:** 1 Department of Virology, Centre Pasteur of Cameroon 567903, Yaoundé, Cameroon; 2 Department of Microbiology, Faculty of Sciences University of Yaounde I 107751, Yaoundé, Cameroon; 3 Virology Unit, Institut Pasteur du Cambodge, Pasteur Network 533891, Phnom Penh, Cambodia; DOE Joint Genome Institute, Berkeley, California, USA

**Keywords:** human *pegivirus* 2 (HPgV-2), metagenomics, nanopore sequencing

## Abstract

Human *pegivirus* (HPgV) genomes were detected in HCV-infected plasma via nanopore metagenomics. Six nearly complete HPgV-2 genomes were identified. Phylogenetic analysis confirmed HPgV-2 genotype. This study reveals co-infection dynamics, highlights viral diversity, and supports improved diagnostics.

## ANNOUNCEMENT

Human *pegivirus* (HPgV), a member of the *Pegivirus* genus within the *Flaviviridae* family (1), is classified into two distinct types: HPgV-1 and HPgV-2 (2, 3). HPgV-1, formerly known as GB virus C (GBV-C) or hepatitis G virus, is the most common virus. It is a 9.3-kb single-stranded, positive-sense RNA virus capable of persistent infection, although it has not been associated with hepatitis or other clinical symptoms or disease (4). HPgV-2 is less common and frequently detected in individuals with hepatitis C virus infection (5); however, it is also not known to cause liver disease (6).

HPgV-2 is a recently identified virus and is genetically distant from HPgV-1, sharing less than 32% amino acid identity with related *pegiviruses* found in rodents and bats (7, 8). HPgV-2 infections have been reported in several countries, including the United States, United Kingdom, Germany, Iran, and China (7–14). Despite its global distribution, data on HPgV-2 remain limited in Cameroon. Furthermore, no complete genome is available from Africa. The present study aimed to fill this gap by characterizing HPgV-2 using complete genomic data from HCV-co-infected individuals in Cameroon.

**Fig 1:**
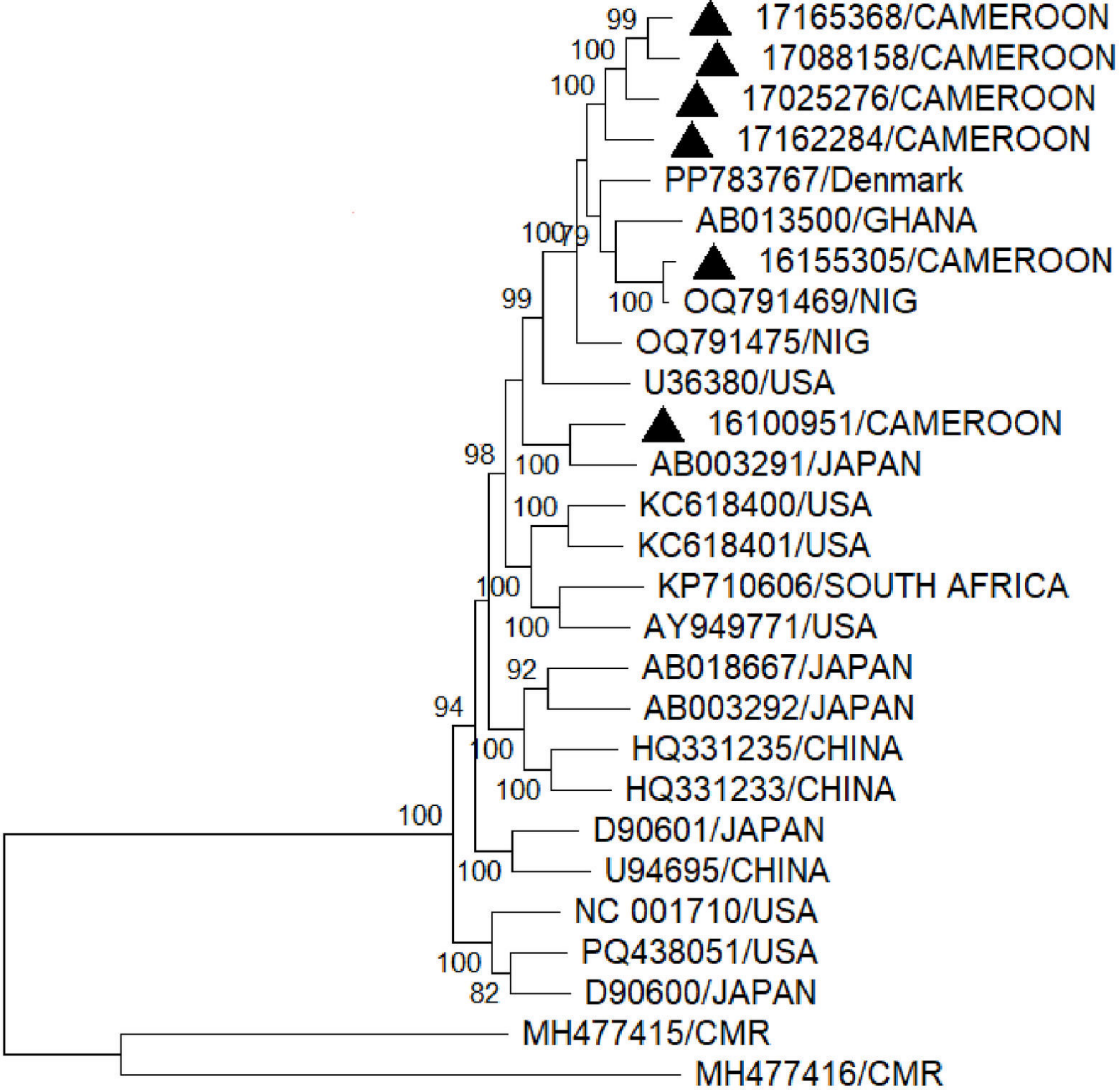
Phylogenetic tree based on the nearly complete genomes of HPgV-2 strains available in GenBank. The accession number and country of origin are indicated for each HPgV-2 strain used in the phylogenetic analysis. Strains identified in this study are marked with a black triangle (▲). Numbers along the branches indicate bootstrap values. A bootstrap analysis with 1000 replicates was performed, and only bootstrap values greater than 70% are shown. The multiple sequence alignment was performed using MUSCLE as implemented in MEGA 12. The tree was constructed using the maximum likelihood method and the Kimura 2-parameter model. GenBank was accessed in April 2024.

Approved by the local ethics committee (CRERSH/C E00571/CERSH/C/2023), this study presents the genomic characterization of human *pegivirus* 2 (HPgV-2) genomes from Central Africa. It is based on a retrospective cohort of 100 plasma samples that tested positive for HCV RNA using the Abbott RealTime HCV assay (Abbott Molecular, USA), with a viral load >100 IU/mL, collected in Yaoundé, Cameroon, between 2013 and 2023. Metagenomic analysis identified six HPgV-2 co-infections. Then, 200 µL of plasma was extracted from each sample using Quick-DNA/RNA pathogen miniprep kit (Zymo Research, California, USA) and eluted in 50 µL of Dnase/Rnase-Free water, and viral genomes were sequenced using Sequence-Independent, Single-Primer Amplification (SISPA) (15) method on the Oxford Nanopore platform (GridIon, R10.4.1 flow cells); purified nucleic acids were cleaned using RNA Clean XP beads (Beckman Coulter, California, USA) prior to complementary DNA (cDNA) synthesis with SuperScript IV Reverse Transcriptase (Thermo Fisher Scientific, USA). Reverse transcription was performed using a nanomer-linked primer, Sol-PrimerA (5′-GTTTCCCACTGGAGGATA-N9-3'), designed to randomly anneal to nucleic acid templates. Second-strand synthesis was subsequently carried out using Sequenase DNA polymerase (Thermo Fisher Scientific, USA). The resulting double-stranded cDNA was amplified by PCR with Phusion High-Fidelity DNA polymerase (ThermoFisher Scientific, USA) and Sol-PrimerB (5′-GTTTCCCACTGGAGGATA-3′), as previously described (15). SISPA amplicon libraries were then constructed using the Native Barcoding Kit (SQK-NBD114.24) and sequenced on an R10.4.1 flow cell (FLO-MIN114; Oxford Nanopore Technologies, Oxford Science Park, UK) in accordance with the manufacturer’s protocol. Subsequent bioinformatic processing was performed as follows: The cleaned reads were taxonomically classified with Kraken2 (v2.1.3) against the MiniKraken2 database (April 2019 version), and the results were visualized using Krona (DOI: 10.1186/1471-2105-12-385). The viral reads were extracted using KrakenTools (DOI: 10.1186/s13059-019-1891-0) and underwent *de novo* assembly with MEGAHIT (v1.2.9) (DOI: 10.1093/bioinformatics/btv033). For each sample, the longest contig generated was aligned against the NCBI nucleotide database via BLASTn (DOI: 10.1016/S0022-2836 (05)80360-2). The most closely matching viral reference sequence was retrieved and used for scaffolding with the contigs generated using the assembly.py script in the viral-ngs environment (https://github.com/broadinstitute/viral-ngs [16]). Gaps in the scaffold were filled by referencing the selected NCBI reference sequence. This refined hybrid scaffold served as the reference for mapping reads: the cleaned reads were aligned with MiniMap2 (v2.24), and the resulting alignment was polished using Medaka (v1.7.2) to generate a final consensus sequence. The depth and extent of genome coverage were then calculated from the polished alignment. This process yielded six high-quality, nearly complete HPgV-2 genomes (approximately 9,000 bp each), with each sequence covering >90% of the length of its closed complete reference genome in GenBank, an average depth of 122×, and a GC content of 58.5% (17). All sequences showed greater than 90% nucleotide similarity to known HPgV-2 reference sequences as determined by BLASTn (NCBI, accessed April 2024), and their detection in multiple patients confirms active local circulation. Notably, phylogenetic analysis revealed tight clustering with global strains, indicating significant genomic conservation worldwide (Fig. 1). The characteristics of six consensus sequences are provided in (Table 1).

**TABLE 1:** Characterization of the six HPgV-2 genomes identified

Accession number	Year of collection	Total reads	G+C content	Genome length (bp)	Average depth (×)	Closest GenBank hit	% Similarity	Source country/year of sampling	SRA accession
PX313739	2016	714,247	59.6%	8,966	140	AB003291	91.69%	Japan/1997	SRX31829865
PX693666	2016	601,245	59.7%	9,386	110	OQ791469	98.38%	Nigeria/2023	SRX31829866
PX693667	2017	632,174	59.6%	9,381	120	KP710599	96.63%	Nigeria/2017	SRX31829867
PX693668	2017	526,451	59.5%	9,378	100	KP710599	95.81%	USA/2015	SRX31829868
PX694297	2017	425,158	59.5%	9,371	80	KP710598	95.94%	USA/2015	SRX31829869
PX694298	2017	584,578	59.4%	9,387	110	KP710599	98.75%	USA/2015	SRX31829870

In conclusion, this study not only describes a rare virus but also demonstrates the utility of proactive genomic surveillance in Africa. It fills a critical gap in global knowledge while strengthening local capacities and providing essential data for regional public health.

## Data Availability

The near-complete genome sequences have been deposited in the GenBank database under accession nos. PX313739, PX693666, PX693667, PX693668, PX694297, and PX694298. The raw NGS reads have been published in the European Nucleotide Archive (BioSamples) under the SRA accession nos. SRX31829865, SRX31829866, SRX31829867, SRX31829868, SRX31829869, and SRX31829870.
